# The African urban food environment framework for creating healthy nutrition policy and interventions in urban Africa

**DOI:** 10.1371/journal.pone.0249621

**Published:** 2021-04-22

**Authors:** Hibbah Araba Osei-Kwasi, Amos Laar, Francis Zotor, Rebecca Pradeilles, Richmond Aryeetey, Mark Green, Paula Griffiths, Robert Akparibo, Milkah Njeri Wanjohi, Emily Rousham, Amy Barnes, Andrew Booth, Kobby Mensah, Gershim Asiki, Elizabeth Kimani-Murage, Nicolas Bricas, Michelle Holdsworth

**Affiliations:** 1 Department of Geography, University of Sheffield, Sheffield, United Kingdom; 2 Department of Population, Family and Reproductive Health, School of Public Health, University of Ghana, Accra, Ghana; 3 Department of Family and Community Health, School of Public Health, University of Health and Allied Sciences, Ho, Ghana; 4 School of Sport, Exercise and Health Sciences, Loughborough University, Loughborough, United Kingdom; 5 Department of Geography & Planning, University of Liverpool, Liverpool, United Kingdom; 6 School of Health and Related Research-ScHARR, University of Sheffield, Sheffield, United Kingdom; 7 African Population and Health Research Center, Nairobi, Kenya; 8 University of Ghana Business school, Accra, Ghana; 9 UMR MoISA (Montpellier Interdisciplinary centre on Sustainable Agri-food systems), (Univ Montpellier, CIRAD, CIHEAM-IAMM, INRAE, Institut Agro, IRD), Montpellier, France; University of Cape Coast, GHANA

## Abstract

This study developed, validated, and evaluated a framework of factors influencing dietary behaviours in urban African food environments, to inform research prioritisation and intervention development in Africa. A multi-component methodology, drawing on concept mapping, was employed to construct a framework of factors influencing dietary behaviours in urban Africa. The framework adapted a widely used socio-ecological model (developed in a high-income country context) and was developed using a mixed-methods research approach that comprised: i. Evidence synthesis consisting of a systematic review of 39 papers covering 14 African countries; ii. Qualitative interview data collected for adolescents and adults (n = 144) using photovoice in urban Ghana and Kenya; and iii. Consultation with interdisciplinary African experts (n = 71) from 27 countries, who contributed to at least one step of the framework (creation, validation/evaluation, finalisation). The final framework included 103 factors influencing dietary behaviours. Experts identified the factors influencing dietary behaviours across all the four levels of the food environment i.e. the individual, social, physical and macro levels. Nearly half (n = 48) were individual-level factors and just under a quarter (n = 26) were at the macro environmental level. Fewer factors associated with social (n = 15) and physical (14) environments were identified. At the macro level, the factors ranked as most important were food prices, cultural beliefs and seasonality. Factors ranked as important at the social level were household composition, family food habits and dietary practices. The type of food available in the neighbourhood and convenience were seen as important at the physical level, while individual food habits, food preferences and socioeconomic status were ranked highly at the individual level. About half of the factors (n = 54) overlap with those reported in an existing socio-ecological food environment framework developed in a high-income country context. A further 49 factors were identified that were not reported in the selected high-income country framework, underlining the importance of contextualisation. Our conceptual framework offers a useful tool for research to understand dietary transitions in urban African adolescents and adults, as well as identification of factors to intervene when promoting healthy nutritious diets to prevent multiple forms of malnutrition.

## Introduction

Africa is experiencing a nutrition transition [1]. Emerging evidence points to a shift in dietary habits [2] linked with urban demographic change [3, 4]. The overall population of the African continent is estimated at 1.3 billion, which is expected to rapidly increase to 2.5 billion by 2050. Almost 44% of Africa’s population is urban, estimated to increase to almost 60% by 2050 [5]. As a consequence, obesity and nutrition-related non-communicable diseases (NR-NCDs), such as obesity, type 2 diabetes, cardiovascular diseases and certain cancers are rapidly increasing among adults and children [6] and becoming a challenging public health problem, especially in urban areas [7]. Undernutrition (especially stunting for children and micronutrients deficiencies) still persists in urban contexts and solutions are therefore needed that tackle multiple burdens of malnutrition. To date, policy responses to NCDs in Africa have either not been prioritised, implemented or achieved much success [8, 9], especially given competition with undernutrition for resources. This lack of success is partly due to the fact that policy interventions have either been designed in, or adapted from high-income countries; such policies are unlikely to be entirely relevant to African settings [10]. Healthy food environments have the potential to prevent multiple forms of malnutrition by targeting shared drivers and promoting healthier diets [11].

Factors that drive food consumption in Africa are poorly understood. In particular, the roles played by people’s social (e.g. family or peer groups) and physical environments (neighbourhoods that individuals live in, e.g. access to fast food outlets) are poorly understood [12, 13]. Stronger evidence is therefore needed in order to characterise the environments in which people live, and how these drive their dietary behaviours. A better understanding of the context can inform smarter policies and interventions to promote healthy food environments, and therefore healthier food consumption by populations in African cities.

Ecological frameworks can provide a useful guide for research and intervention efforts related to dietary behaviours. These frameworks emphasize multi-level linkages (e.g. across individual, social, physical and macro-level environments) [14, 15]. However, existing frameworks of dietary behaviours and food environments [16, 17] tend to be based on evidence from high income countries and need to be adapted to address the context of populations in low-and middle-income country settings. Contextual differences across settings may limit transferability of existing frameworks to the specific context and dynamic changes occurring in urban Africa.

Conceptual frameworks that take a socio-ecological approach to investigating food environments in low-, middle-, and high-income countries have been developed recently [18, 19]. However, the novel contribution of the framework presented in this manuscript is the explicit in-depth focus on urban African contexts and the identification and validation of a comprehensive list of factors across four environmental levels.

This study aimed to develop and evaluate a contextually-sensitive socio-ecological framework of the factors influencing dietary behaviours in urban African food environments. Such a framework could inform research prioritisation and intervention development in Africa to promote healthier food environments and healthier diets among urban populations in Africa.

## Methods

### Expert input

The project team for developing the framework comprised the 17 co-authors of this paper. Additional experts (n = 54), from academia/research, private sector, government and civil society with varying disciplinary backgrounds including public health, nutrition, agriculture, social protection and social sciences contributed to its development. The experts were drawn from all the sub-regions (North, East, Central, West and Southern Africa) (27 countries) and were involved professionally in careers linked with diet or nutrition. Experts were either members of the African Nutrition Society (ANS) and/or delegates who attended the 8^th^ Africa Nutrition Epidemiology Conference (ANEC 8) held in Addis Ababa, Ethiopia in 2018. At ANEC 8, all conference delegates were invited to the symposium by email. Posters were displayed at vantage points at the venue and announcements were made at plenary sessions. Experts who expressed an interest in participating were provided with information about the study, which outlined its aim and the implications of participation before consent was requested. Each expert was involved in at least one step in the framework development or evaluation process: i. factor generation; ii. framework validation and evaluation; and iii. framework finalisation (Table 1).

**Table 1:** Characteristics of participants involved in developing and evaluating the conceptual framework.

	Preparation and generation of factors	Rating and evaluating of framework
**Participant numbers**	n = 20 TACLED and DFC team	n = 71 African Nutrition Society members, African Nutritional Epidemiology Conference delegates International symposium: n = 48 delegates and n = 1 invited expert
**Fields of expertise**	Public Health Nutrition Anthropology Behavioural nutrition Clinical Nutrition Consumer science Dietetics Economics	Food Science Geography/demography Health promotion Nutritional epidemiology Physical activity Statistics
**Countries (n = 27)**	***North Africa*** Algeria Egypt Libya Morocco ***East Africa*** Burundi Ethiopia Kenya Malawi Mayotte Mozambique Rwanda Réunion Tanzania Uganda Zambia Zimbabwe	***West Africa*** Burkina Faso Gambia Ghana Nigeria Saint Helena, Ascension and Tristan da Cunha Senegal ***Central Africa*** Democratic Republic of the Congo ***Southern Africa*** Botswana Lesotho Namibia South Africa

### Working process

**Fig 1:**
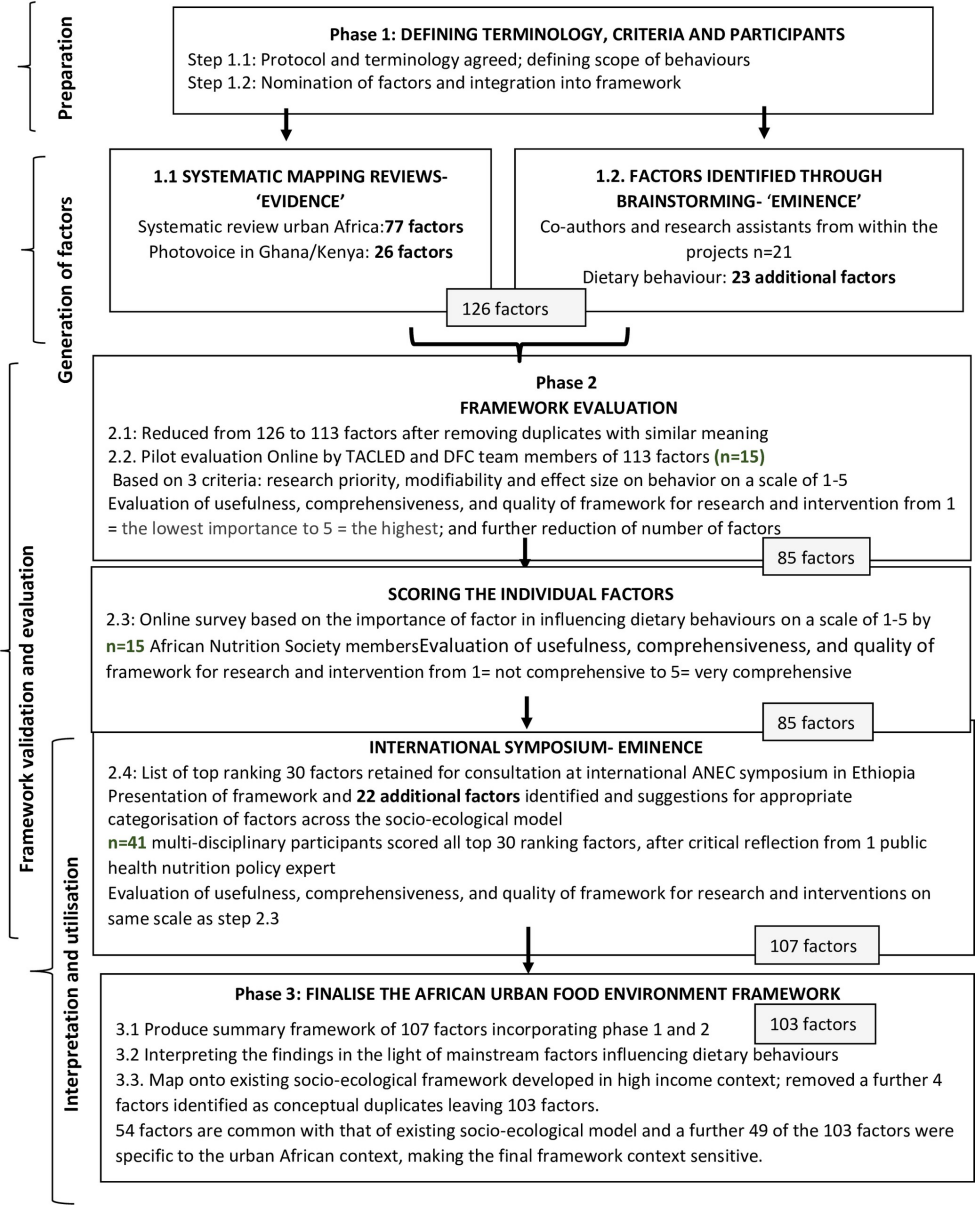
Methodology for developing the African urban food environment framework.

A multi-method approach (Fig 1) was employed in three phases: (1) factor generation, (2) framework evaluation and (3) finalisation, drawing on methods used previously to develop validated frameworks [18, 19]. Framework development drew on primary data collected as part of two projects: ‘Drivers of Food Choice’ (DFC) and ‘Dietary transitions in African cities’: leveraging evidence for interventions and policy to prevent diet-related non-communicable diseases’ (TACLED). These two projects received ethical approval from the Ghana Health Service Ethical Review Committee and the Africa Medical Research Foundation/APHRC in Kenya. Ethical approval for the validation and evaluation of the framework was obtained in June 2018 from the School of Health and Related Research (ScHARR) Ethics Committee, University of Sheffield, UK (reference no 020677). Experts who expressed an interest in participating were provided with information about the study outlining its aim and implications of participation. Personal and identifying details of participants responding to the online questionnaire were removed to ensure anonymity.

#### Phase 1-factor generation

In the preparation step, a protocol was developed to guide framework development. Consensus on the terminology to define dietary behaviours and factors driving these was reached by the project team and research assistants (n = 21). Dietary behaviour was defined as food related behaviours and was grouped into three main categories: food choice, eating behaviour and dietary intake that together capture the main dimensions of dietary behaviours [17, 20].

In the factor generation step, we systematically mapped all the identified factors influencing dietary behaviours, using evidence from i. a systematic mapping review; ii. empirical data from a Photovoice activity conducted in Ghana and Kenya; and iii. expert knowledge. These are outlined below.

*Systematic mapping review*. A systematic mapping review [13] (Fig 1- Phase 1.1) of factors associated with dietary behaviours in urban Africa among adolescents and adults (11–70 years) was carried out. The review synthesised data from quantitative and qualitative studies published from 1971 onwards, when the term ‘epidemiological transition’ was first introduced [21]. As the aim of the framework was to identify factors influencing dietary behaviours for research prioritisation, all factors studied were listed and not restricted to those showing statistically significant association [20].

*Qualitative interviews using Photovoice*. A Photovoice activity complemented by face-to-face in-depth interviews was implemented amongst males/females aged ≥13 years (n = 142), living in low-income neighbourhoods of Accra (n = 62), Ho (n = 32) and Nairobi (n = 48). Although our recent systematic review focusing on dietary behaviours in the context of nutrition transition in the two countries revealed similarities in nutrition and health outcomes, the two countries are at different stages of the nutrition transition [22]. Although Ghana’s population is highly urbanized (>50%), only about a quarter of Kenya’s population live in urban settings. Nevertheless, the urban population in Kenya has a food environment with a higher exposure to supermarkets and formal food service than in Ghana [23]. In the same study, although urban dwellers in both countries are exposed to a combination of nutrient-rich and energy-dense foods, urban areas in Ghana have higher density of fruit and vegetable vendors than was observed in an urban Kenyan setting.

*Sampling*. The qualitative study focused on lower wealth groups. A list of all deprived neighbourhoods within Accra, Ho and Nairobi was compiled and one neighbourhood in each city was randomly selected. In Ghana, James Town was selected from a list of poverty endemic areas in Accra and Dome was selected from a list of poor areas in Ho [24]. In Nairobi, Makadara was selected amongst high deprivation areas [25] that were judged to be safe to work in by the research team. Within these neighbourhoods, participants were purposefully sampled using quota sampling based on key characteristics (i.e. age, gender, body mass index, socio-economic level, education level and occupation status). This was to ensure breadth in the range of views, perspectives and environments that participants were exposed to. Recruitment took place through communities, schools and health services. Data collection spanned September 2017 to June 2018. Written informed consent was obtained from adults and assent from legal guardians of participants <18 years.

The format of the Photovoice activity used to collect data was an adaptation of the conventional format proposed by Wang [26]. In this revised version, participants were asked to take five photographs that depict: i. a place where you eat and/or drink; ii. something/situation that makes eating healthily difficult for you; iii. something/situation that makes eating healthily easy for you; iv. something/situation that influences what you eat in your area; and v. a person that influences your food or drink choices in your area.

Participants attended an initiation meeting with the research team, where they were introduced to Photovoice, given training in using the digital cameras provided and had the opportunity to discuss issues relating to ethics and safety. Follow-up interviews were conducted in either English or in local languages. During these interviews, participants told the ‘stories’ of the five photographs they had taken and were asked to provide a short caption to describe their pictures.

The analytical approach was both theory driven, i.e. *a priori* themes were compiled using socio-ecological models of dietary behaviours [16] and *data driven*, i.e. grounded codes emerging from the data for themes not covered in the theoretical framework. All factors identified through the analysis (S1 Table) were listed and included in the framework.

*Expert consultation*. Experts in the field of nutrition working in the African context were consulted (Fig 1-Phase 1.2). Expert knowledge included brainstorming sessions within two project team meetings. Firstly, each nominated factor from the mapping review and Photovoice activity was categorised into a pre-defined environmental level (individual, social, physical or macro)using a pre-existing ecological framework [16]. This was followed by discussion leading to a common understanding of, and agreement on the factors, and the predefined environmental levels into which each should be placed. This process was also used to discuss the meaning and clarity of terminologies used in the framework. Factors that had not been identified from the mapping review and Photovoice, but were viewed as important were included. In a subsequent project meeting, all factors that were agreed on at the previously identified were presented and the project team repeated the consensus process to agree on factors and the pre-defined levels into which each factor was placed.

#### Phase 2-framework validation and evaluation

The Framework was validated at a meeting involving external stakeholders (Fig 1- Phases 2.1–2.4). The meeting solicited participants’ opinions on what constituted priority factors of dietary behaviours for research, policy and interventions. Additionally, participants appraised the content validity of the framework, i.e. the degree to which it was complete and useful for research and intervention planning, using methods applied previously to validate frameworks [17].

The validation process involved two main tasks that were achieved through rating of factors via an online survey by ANS members (n = 30) and also a face-to-face session involving 41 participants who attended a symposium during the ANEC 8 conference:

1. ***Online survey by*** African Nutrition Society ***(ANS) members*:** Prior to sending the survey to members of the ANS, a pilot exercise involving the project team (Fig 1-Phase 2.2) was conducted in which the criteria used in the development of previous validated frameworks [18, 19] were used to validate the current framework. In doing this, consensus was reached within the group to use only one criterion, i.e. *the overall importance of the factor in influencing dietary behaviours*.
  A revised survey was sent out to all members (n = 400) of the ANS via an email, inviting them to provide scores for all factors included in the framework (Fig 1-Phase 2.3). The survey participants (n = 15) who participated were requested to score factors according to their importance in influencing dietary behaviours in African cities, using a scale of 1 to 5 (where 1 = the lowest importance and 5 = the highest). Participants were also requested to classify factors into the four levels of the ecological framework (individual, social, physical and macro environment). Participants then assessed the content validity of the framework by evaluating its comprehensiveness (completeness) and usefulness for research and interventions in Africa (S1 File). Additionally, participants listed important factors influencing dietary behaviour in African cities that were not included in the list presented in the survey, to ensure that the final framework included all known factors that influenced dietary behaviours in African cities.
2. ***Rating and consultation at ANEC 8 conference symposium-***The second rating exercise (Fig 1-phase 2.4) was undertaken during a symposium at the ANEC 8 conference in Ethiopia (2018) to gain further insights from nutrition researchers and practitioners working in Africa. Registered delegates attending the symposium were invited via an email (n = 350) to attend the workshop. The participants (n = 41) who participated, provided their rating scores on the importance of the top 30 factors that emerged from the online survey influencing dietary behaviours in African cities (Table 2). A pragmatic decision was made to score only the top 30 factors, given the limited period allocated for the symposium during the conference. The face-to-face discussions that accompanied the ratings shed light on the reasons for the ratings. Participants contributed to the discussion and identified knowledge gaps and other factors that they suggested should be added to the framework. The framework was evaluated by participants for its usefulness for research and interventions, and for its comprehensiveness, on a subjective scale of 1 to 5 (where 1 = not comprehensive at all, 2 = not comprehensive, 3 = unable to judge, 4 = quite comprehensive and 5 = very comprehensive).

**Table 2:** Ranking of top 30 dietary factors.

Factor	Importance on dietary behaviour Mean (SD)	Socioecological level
Food prices	4.61(0.78)	Macro
Food habits	4.55(0.60)	Individual
Family food habits & practices	4.39(0.89)	Social
Food preferences	4.35(1.03)	Individual
Household composition	4.30(0.70)	Social
Type of food available	4.17(0.72)	Physical
Convenience (time/effort)	4.17(0.89)	Physical
Cultural beliefs	4.17(1.30)	Macro
Seasonality	4.14(1.13)	Macro
Socio-economic status	4.13(0.92)	Individual
Peer/friend influence	4.13(0.87)	Social
Household food expenditure	4.09(0.67)	Individual
Household food insecurity	4.09(0.67))	Individual
Pregnancy or lactation	4.09(1.20)	Individual
Family influence	4.04(0.98)	Social
Familiarity with food	4.00(1.14)	Individual
Women’s empowerment	4.00(1.04)	Individual
Physical health	4.00(1.04)	Individual
Distance to food outlet	4.00(0.80)	Physical
processed/easy to prepare meals	4.00(0.80)	Macro
Taste	3.96(1.15)	Individual
Time constraints	3.96(0.71)	Individual
Eating at home	3.96(1.11)	Individual
Age	3.96(1.26)	Individual
Food allocation	3.95(0.84)	Social
Food and drink advertising	3.95(1.02)	Macro
Portion size	3.90(1.02)	Individual
Hunger and satiety	3.87(0.97)	Individual
Cooking skills	3.87(1.01)	Individual
Area deprivation	3.87(1.06)	Physical

The scores of the individually rated factors were collated to develop the ranking for all factors and within each environmental level of the framework (i.e. individual, social, physical and macro environments). Means (SD) were calculated as the total sum of the score for each factor divided by the number of scores for each factor.

#### Phase 3: Framework finalisation

The third phase of the framework development process involved finalising and visualising the framework, as well as analysing and comparing the final framework to the existing socio-ecological framework (See Fig 1, Phase 3.1–3.3). This comparative process involved identifying similarities and differences. Two of the co-authors (HOK/MH) independently reviewed the factors in the existing socioecological framework and the current framework, listing the common factors as well as the additional ones that had emerged from the current framework. This step led to the visualisation of the shared factors separately from the additional factors identified in the current framework. Findings were then interpreted in light of existing literature on factors influencing dietary behaviours.

## Results

### Phase 1

In total, 77 factors influencing dietary behaviours were identified from the systematic mapping review, with two-thirds at the individual level (45/77). Factors in the social- (11/77), physical- (12/77) and macro- (9/77) level environments were less frequently identified. Photovoice identified 26 additional factors (S1 Table). An additional 23 factors were identified by experts. At the end of phase 1, after brainstorming within the team, the draft framework consisted of 126 factors. Two of the Authors (HO-K/MH) systematically reviewed the factors to remove duplicates and overlaps, which resulted in a draft framework of 113 factors.

### Phase 2

In phase 2.3 of Fig 1, based on the online survey, the project team further refined the wording of some factors, combining similar factors (e.g., socioeconomic status was combined with class; dependency ratio was changed to household composition because it was deemed as a difficult concept), so that 28 factors were removed, leading to a framework with 85 factors (S2 Table). After the symposium at the ANEC conference, (Fig 1, phase 2.4), 22 additional factors were identified for appropriate categorisation across the socio-ecological framework resulting in 107 factors.

From the results of the online survey (based on all factors) and the ANEC symposium (which reviewed the top 30 factors from the survey), the quality of the framework was judged to be either ‘quite/very comprehensive’ by three-quarters (n = 62) of participants. Around two-thirds of participants indicated that they would consider using the framework for developing interventions (n = 57) or research (n = 46).

The top 10 most important factors that the stakeholders identified as influencing dietary behaviours in urban Africa were ranked as follows: food prices, food habits, family food habits and practices, food preferences, household composition, type of food available in the neighbourhood, convenience (in relation to time/effort), cultural beliefs, seasonality and socio-economic status. The 10 factors that had the lowest scores out of the 103 factors were smoking, road safety, texture, market structures, stress, land use, food labelling, mental health, speed eating and community spaces.

### Phase 3

**Fig 2:**
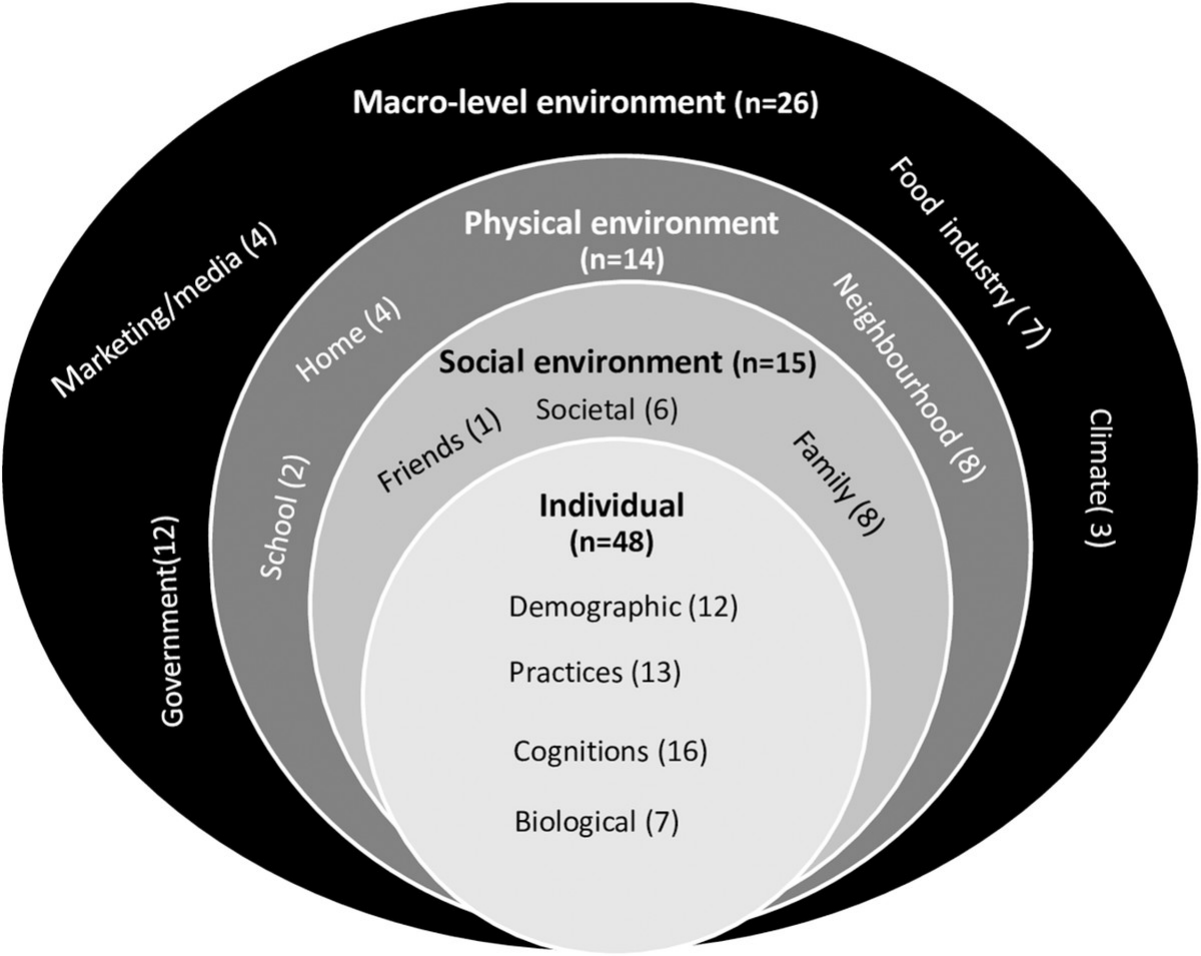
A summary of factors (n = 103) at the different four environmental levels in the African urban food environment framework. The total number of factors identified for each environmental level are shown, e.g. 15 for social environment. The subcategories are then identified with the number of factors within those, e.g. friends, societal, family.

**Fig 3:**
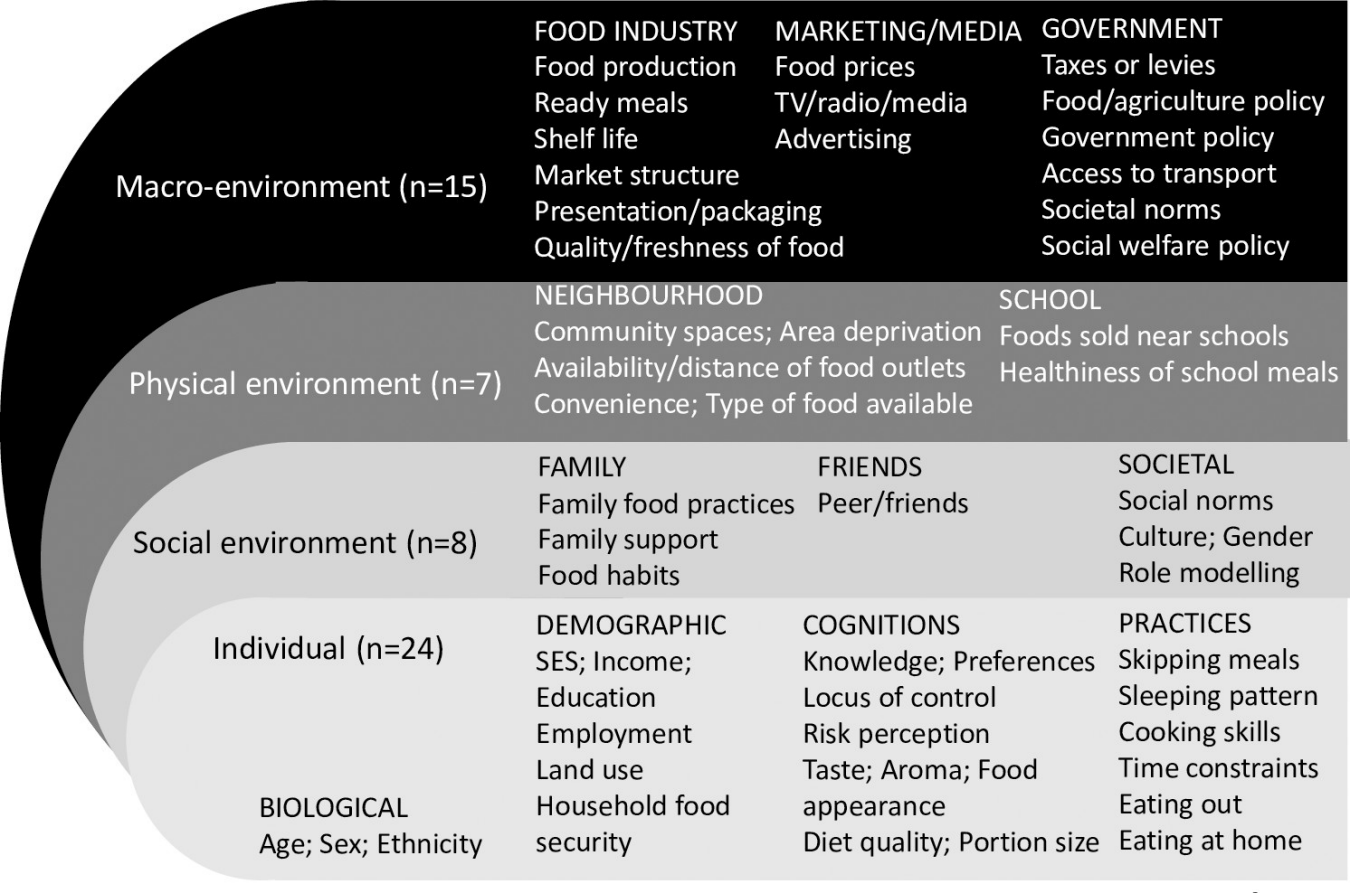
Shared factors (n = 54) in Africa with high income country framework [17].

**Fig 4:**
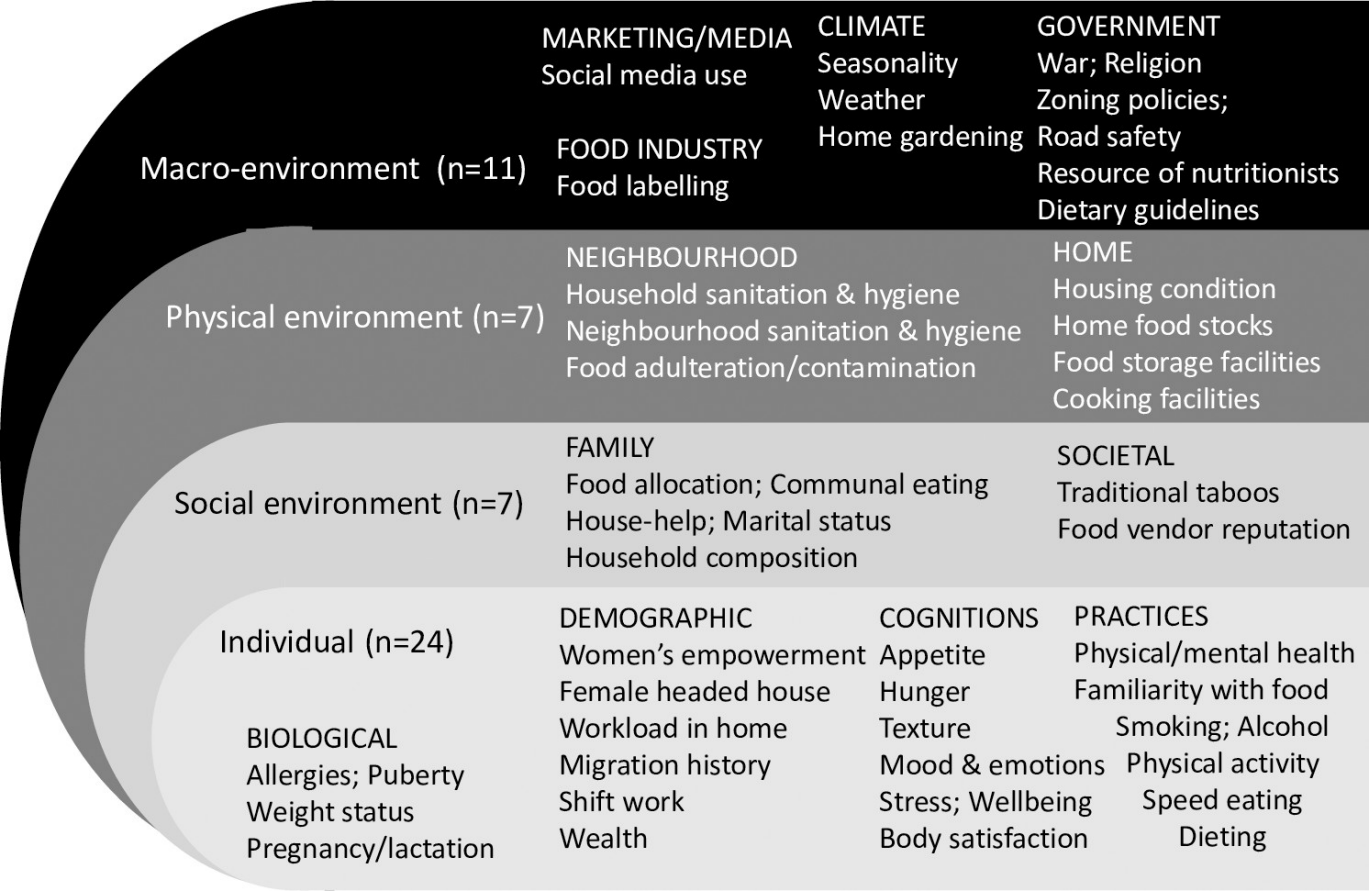
Additional factors (n = 49) that emerged in African urban food environment.

The final framework (Fig 2) consisted of 103 factors after 4 duplicates were removed from the 107 factors identified in phase 2; these comprised 48 individual level factors; 15 social level factors; 14 physical level factors, and 26 macro level factors. Among these factors, 54 (Fig 3) are common with an existing socio-ecological model developed from high-income countries and a further 49 (Fig 4) of the 103 factors emerged as specific to the urban African context, making the final framework context-sensitive.

## Discussion

Using a novel mixed-method and participatory research approach, drawing upon a systematic review (n-39 papers for 14 countries), empirical data collected through photovoice (n = 144; aged ≥13 years) and an expert consultation (n = 71 experts across 27 countries), all from within Africa, our study has developed the first context specific framework of factors influencing dietary behaviours in urban African food environments. Referred to as the African Urban Food Environments Framework, it applies an ecological approach that accounts for the multi-dimensional drivers of dietary behaviours that operate at varying levels of the food environment (including individual-, social-, physical- and macro-levels). Our conceptual framework offers a useful tool for research to understand dietary transitions in urban African adolescents and adults, as well as identification of factors to intervene when tackling unhealthy diets and NR-NCDs.

The framework incorporates 103 factors influencing dietary behaviours, with almost half at the individual level (48/103) and a quarter at the macro environmental level (26/103). Factors in the social (15/103) and physical (14/103) environments were less reported. Of the 103 factors identified, 54 are also found in an existing socio-ecological food environment framework, developed in a high-income context [16]. A further 49 new factors were identified for the urban African context, making our framework context-sensitive, and therefore relevant as a tool to be used in urban Africa.

Factors such as: food prices, convenience, socioeconomic status, taste and hunger emerged as important influences on dietary behaviours in our study, and also feature as important in high income countries [19]. One implication of this is that interventions that address these factors in high income countries could potentially be adapted for use in urban Africa. However, contextual differences must be considered, even when factors are similar, because their degree of relevance, and the way in which they interact with other factors might differ [20, 27] due to fundamental differences in food environments and food procurement practices [19]. For instance, in-kind food transfers and gifts—observed attributes of the African food environment [19], is practiced rarely in high-income countries Also, different contexts may demonstrate varying socio-economic gradients in diets related to risk of obesity in urban areas with varying levels of economic development [28].

Diverse factors could be targeted to improve dietary behaviours among adults in urban Africa. These factors cut across the four levels of the ecological framework. Although the highest number of factors were individual level factors, the most highly ranked factor for importance was food price (a macro-level factor). This is unsurprising because food price volatility in African settings is common and well documented [29]. Instability in food price is an important source of risk, particularly for poor households in sub-Saharan Africa [29]. When food prices change unpredictably, low income urban dwellers who depend on the market for food are particularly affected and their initial response is to cut back on more expensive nutrient-dense foods [29–32]. The type of food available was the 6^th^ prioritised factor, which has also been widely reported as a key factor influencing food consumption. For example, one study that explored the drivers of food availability in Sub-Saharan Africa found that crop production contributed 60% of food availability as compared to off-farm sources [33], and is influenced by seasonality. Indeed, in Sub-Saharan Africa, crop production is dependent on small-farmer subsistence agriculture and rainfall [34]. This situation, combined with high population density, limited capacity to mitigate the effects of natural disasters and poor opportunities for food preservation and storage can influence food availability [35]. This highlights the need to tackle the Global syndemic in African countries of the co-occurrence of undernutrition, obesity and climate change [36].

The importance of factors in the macro level environment on food consumption implies that conventional approaches focussing on individual level behaviour change are insufficient to improve dietary behaviours in urban Africa [13]. Furthermore, evidence shows that upstream interventions, such as fiscal policy are more effective at tackling health outcomes and narrowing inequalities [37]. The role of fiscal policies such as imposing taxes on unhealthy foods and subsidies on healthy food in health promotion in general, and on preventing NCDs have been proposed [38, 39].

Studies have shown that foods high in energy, sugar and fat provide calories at the lowest cost [40] and as a result, families living on a low income may select energy-dense and nutrient-poor packaged processed foods, including those with refined grains, added sugars or high in fat, as a coping mechanism against poverty [41]. To promote healthy diets, policies to reduce the price of healthy foods may be considered in urban Africa. Existing fiscal and regulatory measures that use pricing to make unhealthy foods unattractive, unaffordable, and ultimately unavailable, may be considered [38, 42, 43], although a thorough assessment of the impact of such policies on the urban poor would be necessary prior to introducing legislation. Mandatory food labelling could be introduced to inform consumers about the nutrient content of processed, packaged foods. The WHO regional office for Africa has developed a nutrient profiling model to support countries in the region with the categorisation of food types [44], which would assist this process. However, whilst food labelling emerged as a factor in the macro environment, it was not ranked highly, possibly because of the food industry support required for implementation.

The framework aimed to inform research prioritisation and interventions to promote a healthy, nutritious diets to prevent multiple forms of malnutrition among urban African populations [45]. The framework highlights important factors that need to be considered for intervention development in urban Africa, For instance, addressing the highest rated factors associated with dietary behaviour implies addressing several factors that cut across the four food environment levels. This does not only include individual level factors that have been the focus of earlier dietary interventions [12], but also the physical, social and macro level factors. For example, asking individuals in urban areas in Africa to increase their consumption of raw fruits and vegetables, without taking into account local food hygiene concerns, availability of sustainable fruits and vegetable value chains and conditions and risks for food adulteration, could result in unintended food safety consequences, especially in settings where opportunities to access clean and locally available fruits and vegetables are limited.

Our findings indicate a need for studies that focus on the broader food environment, especially, at the physical- and macro levels. A recent systematic scoping review of food environment research in low- and middle-income countries reported similar findings [46]. Although several factors emerged from the expert consultation in developing this framework, empirical evidence on how the broader food environment, particularly the macro level, influences diet is lacking. The need for research on causal models and pathways of the factors that influence dietary behaviours persists. Future research is needed to deepen our understanding of the inter-relationships between identified factors across the levels, and a holistic systems approach could be valuable. Thus, given the complexity of the nutrition environment, research and evaluation can benefit from an interdisciplinary approach. Future studies need to make direct comparisons between urban and rural populations in Africa to understand differences and commonalities in factors underlying dietary behaviours, so that interventions may be adapted to rural contexts as well. Recent evidence reveals that rural dietary behaviours may also be experiencing nutrition transition [47–49].

The development of the framework has several potential limitations as it incorporated several sources of information, drawing upon a systematic review (39 papers for 14 countries), empirical data collected through photovoice (n = 144; aged ≥13 years) in Ghana and Kenya and an expert consultation (n = 71 experts across 27 countries), all from within Africa. Hence the identified framework is based on several sources of information.

For the systematic review, the strength is it covers factors influencing adolescents and adults’ dietary behaviours in six key databases: EMBASE, MEDLINE, CINAHL, PsycINFO, ASSIA and African Index Medicus. Limitations in terms of the framework development are that the review [13] showed that most research was concentrated in 14 countries and included females, so we have assumed that the findings in these are also relevant to urban contexts in other African countries, as well as to males.

Bias from the photovoice study could also be a consequence of geography, as the studies were conducted in urban poor areas of Ghana and Kenya of adolescents/adults, with a predominance of females. So once again, we are assuming that there is some generalisability from these data to other urban contexts in Africa and to males.

However out consultations with additional experts (n = 54), drawn from all African sub-regions (North, East, Central, West and Southern) helped us reach 27 countries and debate the relevance of the emerging factors from the systematic review and photovoice study for their contexts and for males and females. We should also note that the experts were from a range of professional backgrounds (academia/research, private sector, government and civil society), with varying disciplinary perspectives including public health, nutrition, agriculture, social protection and social sciences, therefore strengthening its validity.

Limitations regarding the use of the socio-ecological model also emerged when developing the framework, as there is overlap between the different environmental levels for some factors. For example, SES pervades all environmental levels. One of the limitations of socio-ecological approaches are that they depict reality as separating individuals from their environment, unlike systems-based approaches, which represent reality better but require data on causality and mechanisms that are often lacking. Socio-ecological models are an easier tool to use to communicate with policy makers and practitioners, compared with systems approaches.

This framework focused on adolescents and adults (aged ≥ 10 years), thus there is still an unmet need for evidence to specifically understand the drivers of food choice in young children and older adults, whose dietary drivers may be different. Developing a framework for the African urban context is a positive first step. However, rather than focusing only on what is different from existing frameworks developed in high income countries, opportunities exist for investigating commonalities. For example, how have factors that drive dietary behaviours been targeted and addressed by interventions in other contexts? Where commonalities exist, there needs to be a focus on whether similar programmes would work because of the different contexts. In this approach, existing interventions should be evaluated in a way that goes beyond ‘what works’, but also identifies ‘for whom it works and in what context’, such as realist approaches [50] to help elucidate the underlying processes.

## Conclusion

The context-appropriate African Urban Food Environment framework draws on published studies, primary data and expert opinion, incorporating insights across Africa through its development, validation, and evaluation. The identification of 49 additional factors in the current framework compared to the high-income country framework reveals the importance of context-specific frameworks. This will support improved healthy dietary behaviours through policies and interventions tailored to the common factors identified to be important in the urban African food environment. Our findings suggest that those interventions likely to succeed need to be multi-level and be tackled by an interdisciplinary team.

## Supporting information

S1 Table Factors from photovoice. (TIF)

S2 Table Socio-ecological framework of factors presented to participants for evaluation (at ANEC 8, 2018). (TIF)

S1 File (DOCX)
