# Supplementary figures and images for: The African urban food environment framework for creating healthy nutrition policy and interventions in urban Africa
Source: PLoS One. 2021 Apr 22;16(4):e0249621. doi: 10.1371/journal.pone.0249621 (PMC8061920; doi:10.1371/journal.pone.0249621)

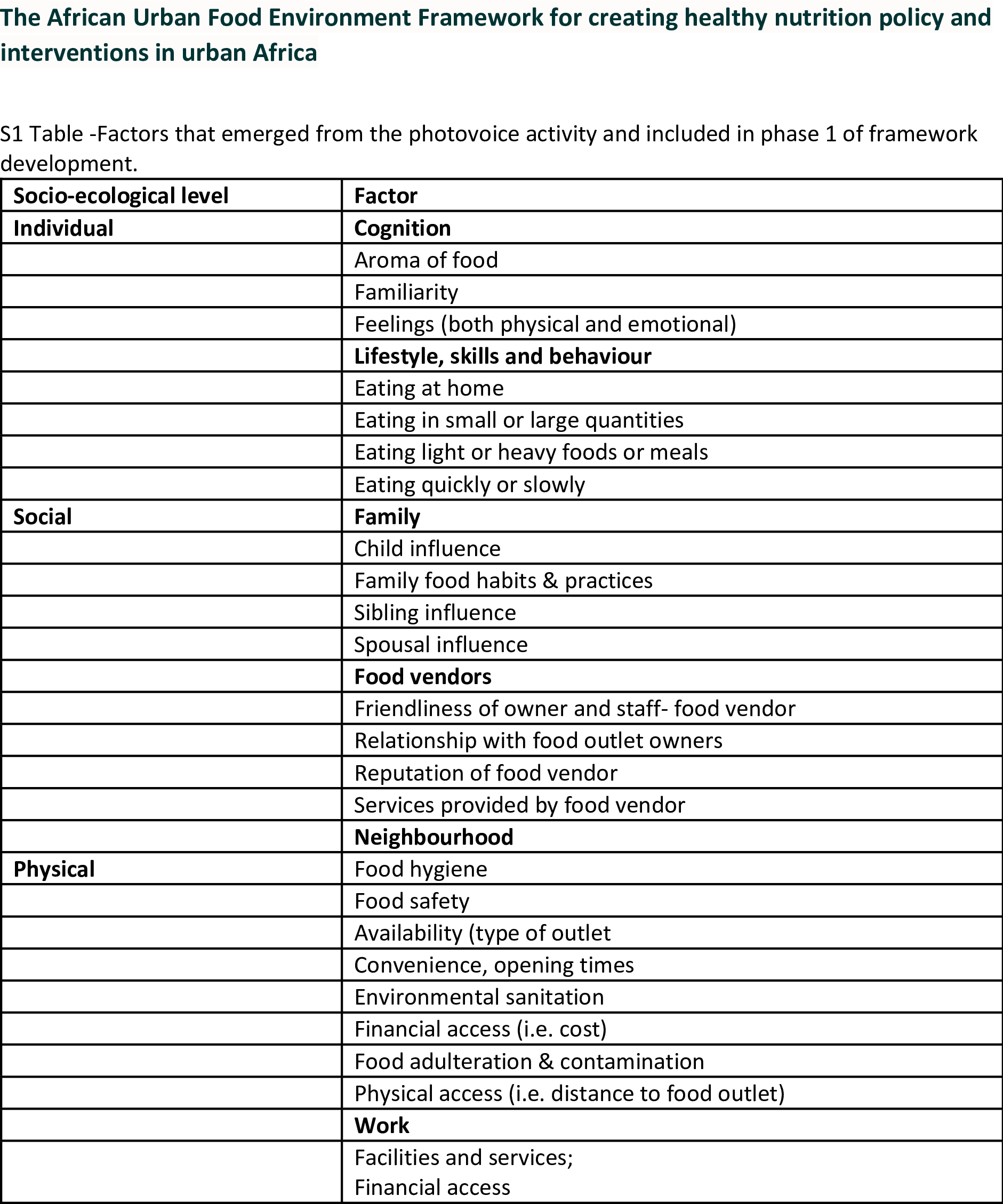

Supplement: S1 Table — (TIF) [file pone.0249621.s001.tif]

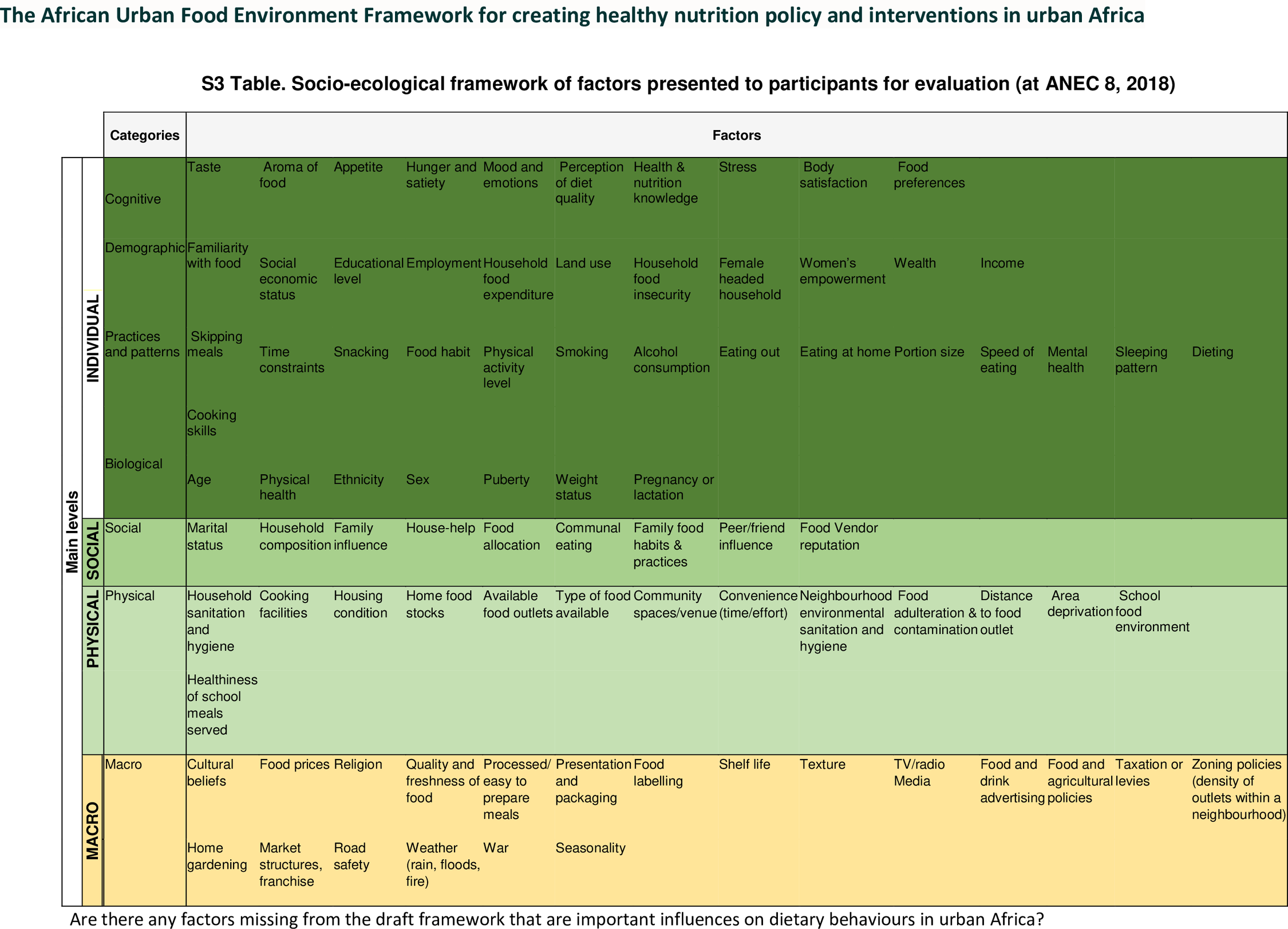

Supplement: S2 Table — (TIF) [file pone.0249621.s002.tif]
