# Supplementary material for: The African urban food environment framework for creating healthy nutrition policy and interventions in urban Africa
Source: PLoS One. 2021 Apr 22;16(4):e0249621. doi: 10.1371/journal.pone.0249621 (PMC8061920; doi:10.1371/journal.pone.0249621)
Supplement: S1 File — (DOCX) [file pone.0249621.s003.docx]

**S1 File. Evaluating a conceptual framework of factors influencing dietary behaviours in the urban food environment**

We have developed a conceptual framework of factors influencing the food environment in urban Africa using evidence from research in Africa and expert opinion.  Building on this, we would like your views on the importance of the different factors on dietary behaviours in African cities. Dietary behaviour is defined here as the manner in which an individual act or performs in relation to diet. This includes food choice, eating behaviour and dietary intake. Many thanks for your time!

**1. Your main disciplines/areas of expertise (please tick a maximum of 3):**

Public Health Nutrition Geography/demography

Anthropology Statistics

Behavioural nutrition Psychology (health, cognitive and social)

Clinical Nutrition Physical activity

Consumer science Nutritional epidemiology

Dietetics Health promotion

Economics Food Science

Other (please specify) _____________________________________

**2. What category does your current employment fall into? Please tick the closest response**

Academic/Research

Food industry

Government department

International organisation, e.g. FAO/WHO

NGO

Private consultancy/ Freelance

Other (please specify) _____________________________________

**3. Which countries in Africa do you work or have most experience of working with? (maximum of 3)**

a. _____________________________b. ________________________c. ________________________

#### 4. Given your experience of nutrition in urban Africa, how comprehensive do you think the framework is? Please tick one

| **Not comprehensive at all** | **Not comprehensive** | **Unable to judge** | **Quite comprehensive** | **Very comprehensive** |
| --- | --- | --- | --- | --- |
| 1 | 2 | 3 | 4 | 5 |

#### 5. Are there any factors missing from the framework that are important influences on dietary intake in urban Africa? Please list: ……………………………………………………………………………………………………………………………………………………………………………………………………………………………………………………………………………………………………………………………………………………………………………………………………………………………...

**6. Please score the following factors in your opinion on their importance in influencing dietary behaviours in African cities. Please use a scale of 1 to 5, where 1= the lowest importance and 5=the highest. Please tick one box per line.**

| **Socio-ecological level** | **Factors** | **Very low importance** | **Low importance** | **Neither low or high** | **High importance** | **Very high importance** |
| --- | --- | --- | --- | --- | --- | --- |
| Individual | Taste | 1 | 2 | 3 | 4 | 5 |
|  | Time constraints | 1 | 2 | 3 | 4 | 5 |
|  | Eating at home | 1 | 2 | 3 | 4 | 5 |
|  | Age | 1 | 2 | 3 | 4 | 5 |
|  | Portion size | 1 | 2 | 3 | 4 | 5 |
|  | Hunger and satiety | 1 | 2 | 3 | 4 | 5 |
|  | Cooking skills | 1 | 2 | 3 | 4 | 5 |
|  | Familiarity with food | 1 | 2 | 3 | 4 | 5 |
|  | Food preferences | 1 | 2 | 3 | 4 | 5 |
|  | Socio-economic status | 1 | 2 | 3 | 4 | 5 |
|  | Household food expenditure | 1 | 2 | 3 | 4 | 5 |
|  | Household food insecurity | 1 | 2 | 3 | 4 | 5 |
|  | Pregnancy or lactation | 1 | 2 | 3 | 4 | 5 |
|  | Women’s empowerment | 1 | 2 | 3 | 4 | 5 |
|  | Physical health | 1 | 2 | 3 | 4 | 5 |
|  | Food habits | 1 | 2 | 3 | 4 | 5 |
| Social | Family eating practices | 1 | 2 | 3 | 4 | 5 |
|  | Family influence | 1 | 2 | 3 | 4 | 5 |
|  | Peer/friend influence | 1 | 2 | 3 | 4 | 5 |
|  | Household composition | 1 | 2 | 3 | 4 | 5 |
|  | Food allocation |  |  |  |  |  |
| Physical | Type of food available | 1 | 2 | 3 | 4 | 5 |
|  | Convenience (time/effort) | 1 | 2 | 3 | 4 | 5 |
|  | Distance to food outlet | 1 | 2 | 3 | 4 | 5 |
|  | Area deprivation | 1 | 2 | 3 | 4 | 5 |
| Macro | Cultural beliefs | 1 | 2 | 3 | 4 | 5 |
|  | Seasonality | 1 | 2 | 3 | 4 | 5 |
|  | Processed/easy to prepare | 1 | 2 | 3 | 4 | 5 |
|  | Food prices | 1 | 2 | 3 | 4 | 5 |
|  | Food and drink advertising | 1 | 2 | 3 | 4 | 5 |

#### 7. Would you consider using the framework in your own research? Please tick one

#### Yes No Not applicable (I don’t conduct research)

#### 8. Would you consider using the framework in developing interventions? Please tick one

#### Yes No Not applicable (I don’t conduct interventions)
